# Construction and evaluation of an individualized nomogram prediction model for posterior vitreous detachment in patients with cataract surgery

**DOI:** 10.3389/fmed.2025.1710501

**Published:** 2025-12-03

**Authors:** Jia Yu Tang, Xiao Hong Lyu

**Affiliations:** 1 Queen Mary College, Nanchang University, Nanchang, Jiangxi, China; 2 Nanchang University Affiliated Eye Hospital, Nanchang, Jiangxi, China

**Keywords:** cataract surgery, posterior vitreous detachment, nomogram, prediction model, risk factors

## Abstract

**Objective:**

To analyze factors linked to posterior vitreous detachment (PVD) after cataract surgery and develop a nomogram prediction model.

**Methods:**

A total of 480 cataract patients who underwent phacoemulsification from January 2022 to December 2024 were enrolled. They were divided into modeling (*n* = 240) and validation (*n* = 240) groups. Based on postoperative PVD status, the modeling group included 80 PVD and 160 non-PVD cases, while the validation group had 84 PVD and 156 non-PVD cases. Demographic and clinical data were analyzed. Multivariate logistic regression identified risk factors, and a nomogram was constructed. The model’s performance was evaluated using ROC curves, calibration plots, and decision curve analysis (DCA).

**Results:**

No significant differences were found in gender, BMI, diabetes, hypertension, vitreous cavity depth, preoperative vitreous opacity, or lens nuclear hardness (*P* > 0.05). However, age, axial length, preoperative vitreous liquefaction, cumulative ultrasound energy (CUE) time, and operation time differed significantly (*P* < 0.05). Logistic regression confirmed these as independent predictors of PVD. ROC analysis indicated strong discriminative ability. Calibration curves showed good fit (modeling group: χ^2^ = 9.320, *P* = 0.316; validation group: χ^2^ = 6.282, *P* = 0.616). DCA revealed clinical net benefit when the risk threshold exceeded 0.02.

**Conclusion:**

Older age, longer axial length, greater preoperative vitreous liquefaction, longer CUE time, and extended operation time independently predict PVD after cataract surgery. The nomogram based on these factors shows strong predictive accuracy and clinical utility.

## 1 Introduction

Cataract is a common age-related degenerative eye disease characterized by a gradual decrease in lens transparency. Its incidence rate is increasing year by year with the aging of the global population (1–3). At present, the primary approach to managing cataracts centers on surgical treatment, with phacoemulsification followed by intraocular lens implantation being the most widely adopted technique. In recent years, advancements in technology have led to the development and increasing use of femtosecond laser-assisted cataract surgery, offering a more precise and automated alternative in selected cases. These surgical methods have become the standard treatment for restoring patients’ visual function and improving their quality of life (4, 5). Although the safety and accuracy of surgery have been significantly improved with the continuous advancement of microsurgical techniques and equipment, postoperative complications are still the key factors affecting visual recovery and long-term prognosis. Posterior vitreous detachment (PVD) represents a frequent structural alteration observed following cataract surgery. The fundamental pathological mechanism mainly entails the physical separation of the posterior vitreous cortex from the inner boundary of the retina (6). Cataract surgery induces an inflammatory cascade and the buildup of reactive oxygen species, both of which can disrupt the normally stable interactions between collagen fibrils and hyaluronic acid within the vitreous humor. This disruption contributes to the breakdown of the gel-like vitreous structure. Moreover, following surgical intervention, the vitreous cavity may undergo relative expansion, resulting in a localized reduction in the density of collagen and hyaluronic acid. This dilution effect further facilitates vitreous liquefaction and condensation, thereby hastening the development of PVD. Additionally, the energy generated during the procedure, along with alterations in intraocular fluid flow and pressure dynamics, acts synergistically to accelerate the natural progression of PVD (7). This assessment can not only help identify high-risk patients, but also provide a scientific basis for optimizing perioperative management, reducing the incidence of retinal-related complications, and improving surgical safety.

As an efficient, intuitive, and easy-to-use prediction tool, the nomogram has significant application potential in the development of precision medicine and personalized treatment strategies due to its excellent discriminatory ability. The model integrates multiple clinical factors or biological indicators into a quantitative scoring system and displays it in a graphical form, allowing clinicians to easily estimate the risk of disease occurrence, progression, or prognosis for a specific patient (8). Through this intuitive visualization tool, medical staff can more effectively develop personalized intervention measures for patients, thereby optimizing the clinical decision-making process and improving treatment outcomes.

Therefore, through this study, we hope to provide an effective predictive tool for clinical practice to help doctors better assess patients’ individualized risks before, during, and after surgery, thereby providing a basis for postoperative management and improving the overall treatment effect of patients.

## 2 Materials and methods

### 2.1 General information

This study included 480 patients who underwent phacoemulsification cataract surgery at our hospital between January 2022 and December 2024. Sample size estimation was based on a PVD incidence rate of 33%, test power of 80%, and a significance level of α = 0.05. Using PASS 15.0 software, the minimum sample size per group was calculated as 220 cases. To ensure adequate statistical power, the final sample size was set at 480 cases. Grouping was performed using the random number generator within SPSS 21.0 software, randomly assigning patients in a 1:1 ratio to the modeling group (*n* = 240) and the validation group (*n* = 240). According to the patients’ postoperative vitreous detachment, the modeling group was divided into a PVD group (*n* = 80) and a non-PVD group (*n* = 160), and the validation group was divided into a PVD group (*n* = 84) and a non-PVD group (*n* = 156).

The inclusion criteria were: (1) meeting the diagnostic criteria for cataract in the “Chinese Journal of Ophthalmology (3rd Edition)” (9); (2) intraocular pressure ≤ 21 mmHg; (3) all underwent phacoemulsification combined with intraocular lens implantation. Exclusion criteria: (1) Patients with concurrent eye diseases such as retinal detachment, corneal disease, and glaucoma; (2) Patients presenting with complex cataract manifestations such as lens dislocation, nuclear opacity, or hard nucleus; (3) Patients with preoperative PVD; (4) Patients with fundus changes such as choroidal atrophy and posterior scleral staphyloma in the operated eye; (5) Patients with a history of previous eye surgery; (6) Patients with incomplete clinical data or follow-up time of < 1 month. This study was approved by the ethics committee of our hospital.

### 2.2 Methods

Data collection: Using keywords such as cataract and posterior vitreous detachment, search domestic and international databases including CNKI and PubMed. Organize relevant literature, selecting credible studies published within the last 5 years. Analyze pertinent content within these publications to summarize potential risk factors influencing posterior vitreous detachment following cataract surgery. Demographic and baseline clinical characteristics of the participants were gathered, including gender, age, body mass index (BMI), history of diabetes, history of hypertension, axial length (AL), vitreous cavity depth, lens nuclear hardness grade, preoperative vitreous opacity, preoperative vitreous liquefaction, cumulative ultrasound energy (CUE) time, and surgical time.

Diagnostic criteria and follow-up protocol for postoperative PVD: Diagnosis of postoperative PVD shall be independently performed by two experienced retinal specialists. Assessment shall employ combined ocular B-scan ultrasound (10 MHz probe) and optical coherence tomography (OCT), with follow-up scheduled at 1 month postoperatively. PVD is defined as complete separation of the posterior vitreous cortex from the retinal inner limiting membrane, with or without Weiss’s ring.

### 2.3 Statistical methods

This study employed stringent inclusion and exclusion criteria for subject selection. All enrolled patients possessed complete clinical records with no missing critical variables. Data analysis was performed with SPSS version 21.0 computing environment. The Shapiro-Wilk test was applied to assess the normality of variable distributions. All continuous variables in this study conformed to a normal distribution. Normally distributed numerical data are presented as mean ± SD, and differences between independent groups were evaluated using the independent-samples *t*-test. Categorical variables are summarized as percentages, and the chi-square test was used to compare proportions across groups. First, univariate analysis (*t*-tests or chi-square tests) screened for potential risk factors associated with postoperative PVD (*P* < 0.05). Subsequently, variance inflation factors (VIF) were calculated, and highly collinear variables with VIF > 5 were excluded to mitigate multicollinearity interference. The screened variables were incorporated into a multivariate logistic regression model to determine independent factors influencing postoperative PVD, thereby constructing a predictive model. Model performance was assessed through discrimination, calibration, and decision curve analysis. Bilateral *P* < 0.05 was considered statistically significant.

## 3 Results

### 3.1 Comparison of clinical data

The differences in age, AL, preoperative vitreous liquefaction degree, CUE time, and surgical time were significant (*P* < 0.05), as shown in Table 1.

**TABLE 1:** Comparison of baseline data.

Project	PVD group (*n* = 80)	Non-PVD group (*n* = 160)	*t*/χ^2^/*Z*	*P*
Gender	43 (53.75)	84 (52.50)	0.033	0.855
Male				
Female	37 (46.25)	76 (47.50)		
Age [years, *n* (%)]	67 (83.75)	73 (45.63)	31.894	<0.001
>60				
≤60	13 (16.25)	87 (54.37)		
BMI [kg/m^2^, *n* (%)]	39 (48.75)	71 (44.38)	0.411	0.521
<18				
≥18	41 (51.25)	89 (55.62)		
History of diabetes [cases, *n* (%)]	21 (26.25)	49 (30.62)	0.494	0.482
Have				
None	59 (73.75)	111 (69.38)		
History of hypertension [cases, *n* (%)]	61 (75.25)	114 (71.25)	0.675	0.411
Have				
None	19 (23.75)	46 (28.75)		
AL [mm, *n* (%)]	66 (82.50)	70 (43.75)	32.613	<0.001
>25				
≤25	14 (17.50)	90 (56.25)		
Vitreous cavity depth [mm, *n* (%)]	12 (15.00)	21 (13.12)	0.158	0.691
>17				
≤17	68 (85.00)	139 (86.88)		
Lens nuclear hardness grade	26 (32.50)	47 (29.38)	0.246	0.620
I to III				
IV to V	54 (67.50)	113 (70.62)		
Preoperative vitreous opacity	27 (33.75)	0 (31.25)	0.205	0.903
Mild/moderate				
Severe	46 (57.50)	94 (58.75)		
Adhesion	7 (8.75)	16 (10.00)		
Preoperative vitreous liquefaction degree	63 (78.75)	59 (36.88)	37.419	<0.001
> 0%				
≤50%	17 (21.25)	101 (63.12)		
CUE time (min)	2.07 ± 0.33	1.48 ± 0.29	14.110	<0.001
Surgical time (min)	22.54 ± 4.93	20.11 ± 5.06	3.534	<0.001

PVD, posterior vitreous detachment; BMI, body mass index; AL, axial length; CUE time, cumulative ultrasound energy time.

### 3.2 Multivariate logistic regression analysis

The results showed that age, AL, preoperative vitreous liquefaction degree, CUE time, and surgical time were independent influencing factors for the occurrence of postoperative PVD (*P* < 0.05) (Table 2).

**TABLE 2:** Multivariate logistic regression analysis of PVD in patients with cataract surgery.

Variable	*B*	*SE*	Wald	*P*	Odds ratios	95% CI for odds ratios	
						Lower limit	Upper limit
Age	1.936	0.575	11.357	0.001	6.932	2.248	21.374
AL	1.860	0.534	12.128	< 0.001	6.421	2.255	18.285
Preoperative vitreous liquefaction degree	1.764	0.509	12.023	0.001	5.834	2.153	15.809
CUE time	6.519	1.068	37.266	< 0.001	677.844	83.590	5496.749
Surgical time	0.120	0.054	5.030	0.025	1.128	1.015	1.253
Constant	–15.407	2.512	37.614	0	0		

PVD, posterior vitreous detachment; AL, axial length; CUE time, cumulative ultrasound energy time.

### 3.3 Construction of a prediction model for PVD in postoperative cataract patients

**FIGURE 1:**
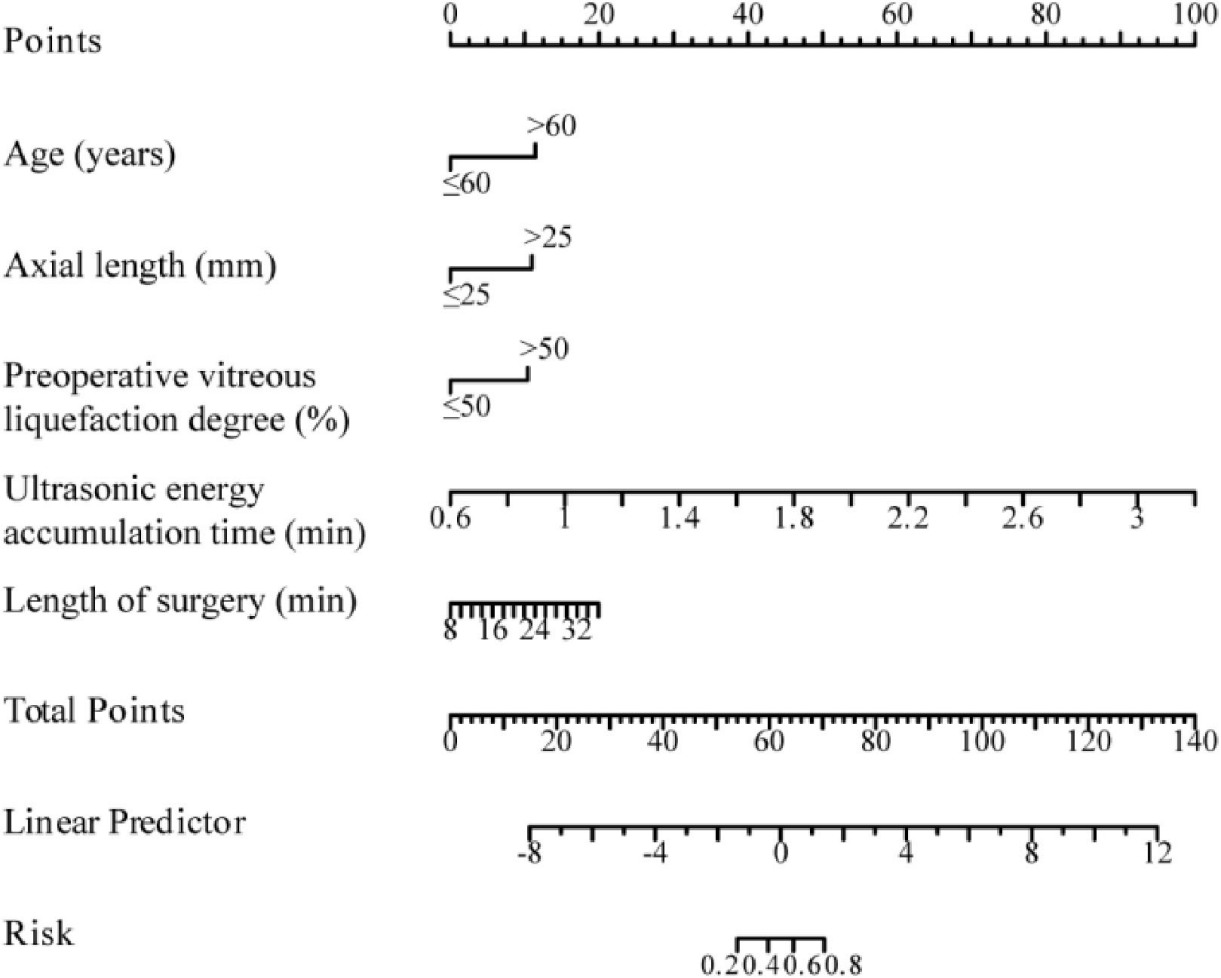
Nomogram of the prediction model for PVD in patients after cataract surgery.

Based on the results of multivariate logistic regression analysis, age, axial length, preoperative vitreous liquefaction degree, CUE time, and surgical time were included in the survival prediction model to construct a nomogram prediction model (Figure 1) to graphically assess and monitor the development of PVD in individuals following surgical intervention.

### 3.4 Effectiveness analysis of prediction model for PVD in patients with cataract surgery

**FIGURE 2:**
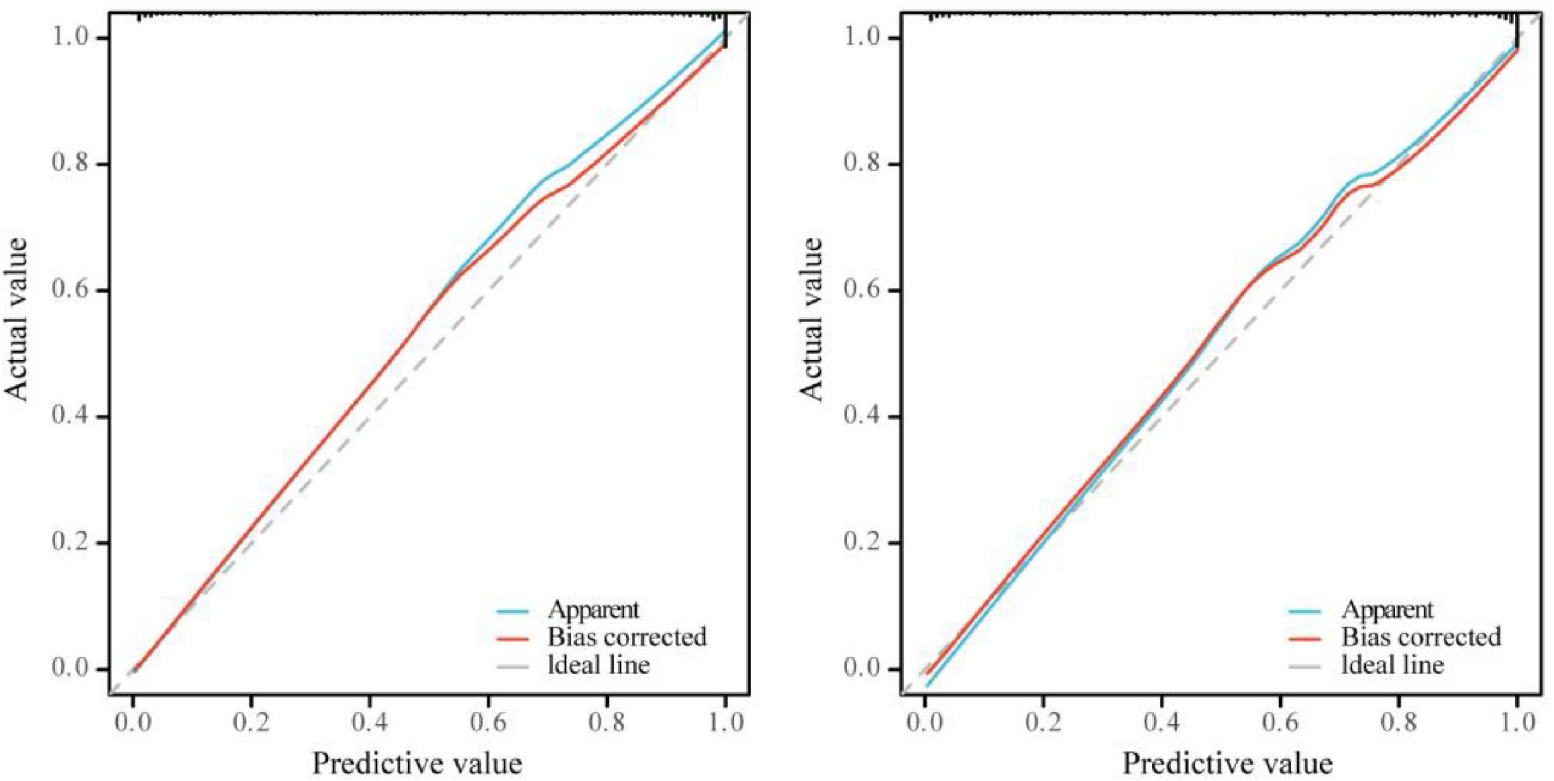
Calibration curve. Left: Modeling group; Right: Validation group.

**FIGURE 3:**
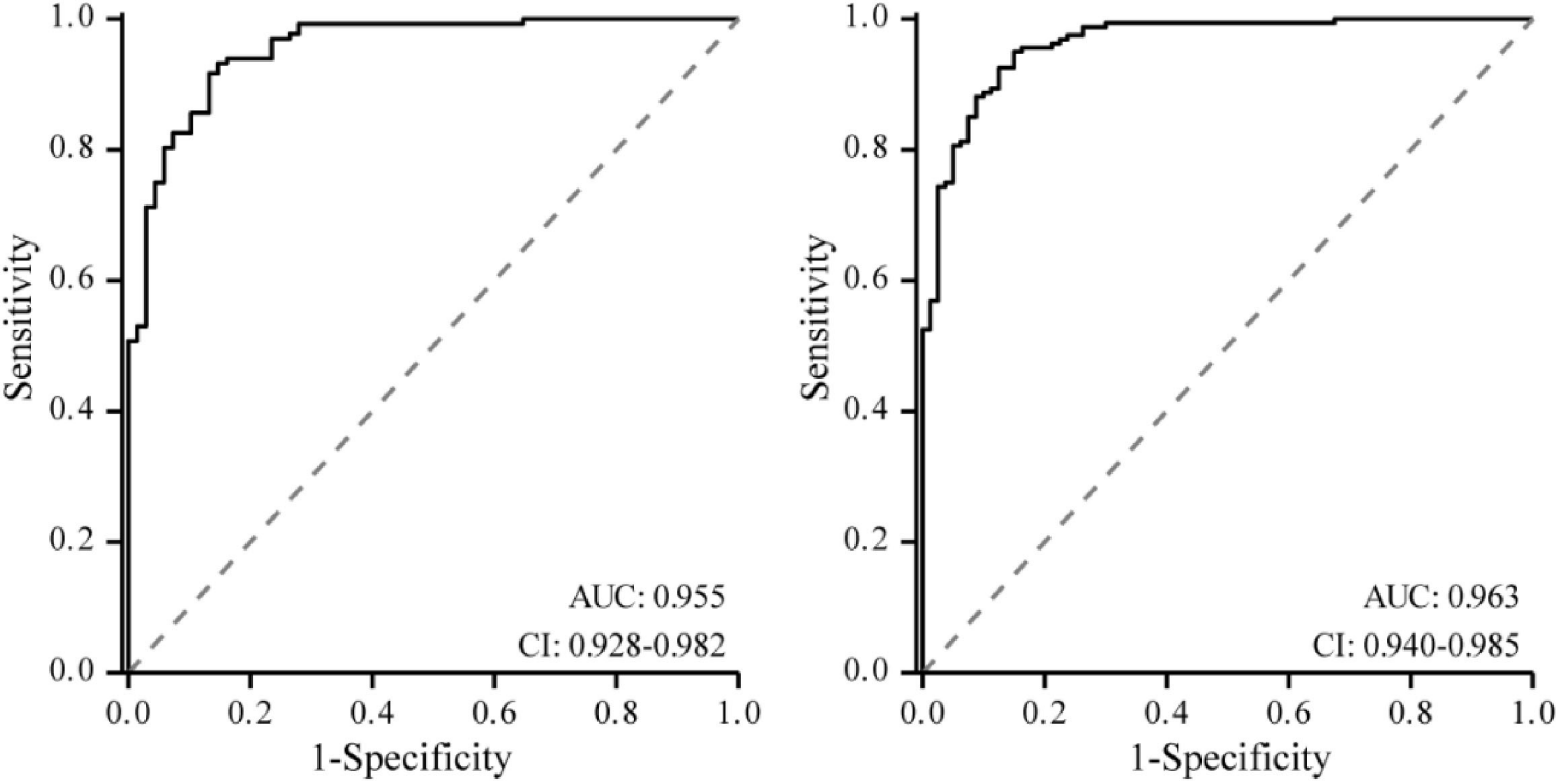
ROC curve. Left: Modeling group; Right: Validation group.

**FIGURE 4:**
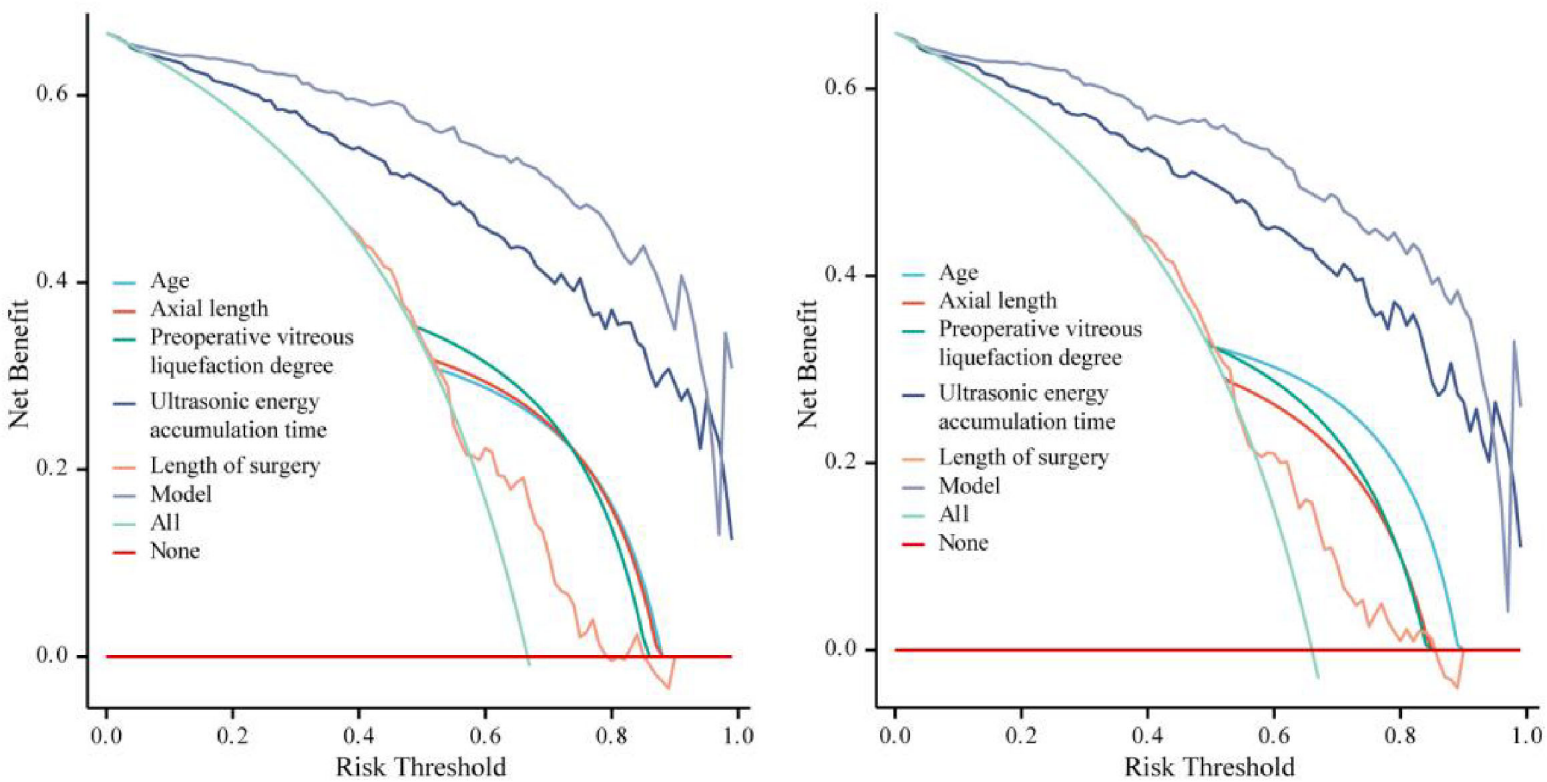
DCA curve. Left: Modeling group; Right: Validation group.

The nomogram prediction model for PVD after cataract surgery was validated. The calibration curve showed that the goodness-of-fit test (HL) in the modeling group was χ^2^_A_ = 9.320, *P*_A_ = 0.316; the C-index was 0.963 (95% *CI*: 0.940–0.985); and the likelihood ratio test was χ^2^_A_ = 194.840, *P*_A_ < 0.05. In the validation group, the HL was χ^2^_B_ = 6.282, *P*_B_ = 0.616; the C-index was 0.955 (95% *CI*: 0.927–0.983); and the likelihood ratio test was χ^2^_B_ = 155.890, *P*_B_ < 0.05 (Figure 2). The ROC curve showed that the AUC of the model in the modeling group was 0.963 (95% *CI*: 0.940–0.985), with a cutoff value of 0.183 and a Youden index of 0.800. In the validation group, the AUC was 0.955 (95% *CI*: 0.928–0.982), with a cutoff value of 0.080 and a Youden index of 0.785 (Figure 3). DCA indicated that the predictive model provided a meaningful net clinical advantage, particularly when the threshold probability was > 0.02 (Figure 4).

## 4 Discussion

As the global population continues to age at an accelerating pace, cataracts have emerged as a leading contributor to visual disability and, in advanced cases, permanent blindness among older adults (10). This condition imposes a considerable burden from an epidemiological perspective, affecting a growing number of individuals and presenting significant challenges to public health systems worldwide. According to data, as of 2020, there were approximately 57.1 million patients with moderate to severe visual impairment worldwide, of which cataracts were the main cause; in addition, approximately 13.4 million cases of blindness were caused by cataracts, highlighting the important position of cataracts in global public health (11, 12). At present, the treatment of cataracts relies on surgical intervention, among which the standard surgical method is phacoemulsification combined with intraocular lens implantation. However, no other non-surgical treatments have been proven to be effective. Although the global cataract surgery rate has continued to rise in the past few decades, there is still a significant gap in surgical coverage in China compared with Europe, the United States and some developed Asian countries, and the uneven distribution of medical resources between regions is still prominent (13, 14). PVD is a frequently observed complication following cataract surgery. Multiple clinical investigations have demonstrated that, in comparison to untreated eyes, eyes undergoing cataract surgery experience a markedly accelerated rate of PVD development. Furthermore, the likelihood of complete PVD occurring within 1 year postoperatively is substantially elevated, with studies reporting a relative risk increase of about sevenfold compared to the control group (15). This suggests that surgical procedures may promote vitreous liquefaction and posterior detachment, thereby increasing the possible likelihood of adverse events including retinal tears and retinal detachment. In light of the background outlined above, the present study was conceived with the aim of retrospectively analyzing and pinpointing factors that independently predict the occurrence of PVD after cataract surgery. By employing multivariate logistic regression analysis, a nomogram was constructed to serve as a predictive instrument, designed to demonstrate high levels of discrimination and reliable calibration in estimating individual patient risk. It is anticipated that this model will facilitate early identification and risk stratification of high-risk individuals, thereby supporting evidence-based clinical decision-making in tailoring postoperative monitoring protocols and refining both preoperative assessments and postoperative care strategies.

The findings derived from multivariate logistic regression analysis indicated that age, axial length, degree of vitreous liquefaction before surgery, CUE time, and operation time were independent risk factors for postoperative PVD. Cataract represents the leading cause of age-related lens opacification among older adults, with its prevalence rising markedly as individuals grow older. A substantial body of epidemiological research has repeatedly confirmed that increasing age is a major contributing factor in the onset and progression of cataracts. It is estimated that approximately 66% of individuals aged 80 years and older are affected by some form of lens opacity, highlighting the strong association between aging and this ocular condition (16). The present research additionally revealed that individuals aged 60 years and above are at a considerably elevated risk of experiencing PVD following cataract surgery when compared to their younger counterparts. Specifically, the incidence of postoperative PVD in patients aged ≥ 60 years is 6.932 times that of patients aged < 60 years, indicating that aging is closely related to the decline in vitreous stability after surgery. In addition, a higher degree of vitreous liquefaction before surgery was identified as another independent risk factor for accelerating the occurrence of postoperative PVD. The vitreous is a transparent gelatinous substance located between the back of the lens and the front of the retina, primarily made up of water, along with collagen fibrils and hyaluronic acid as key structural components. With age, the microstructure of the vitreous gradually undergoes degenerative changes, including the depolymerization of collagen fibers and the degradation of hyaluronic acid, which leads to the transformation of its gel state into a liquid state. This process is called vitreous liquefaction (17). This pathological alteration weakens the attachment between the posterior vitreous boundary layer and the retinal internal surface, facilitating the progressive separation of the posterior vitreous cortex from the underlying retina, and thereby leading to the development of posterior vitreous detachment PVD (18, 19). Therefore, if extensive liquefaction areas or structural instability are found in the vitreous before surgery, it may indicate that the risk of PVD will be further increased during surgery due to changes in perfusion flow dynamics (20). Therefore, preoperative assessment of vitreous condition is not only important for identifying patients at high risk of PVD, but also helps to optimize surgical plans, strengthen postoperative monitoring, and prevent related retinal complications, thus providing important guidance value in clinical practice.

AL is another important independent risk factor for PVD after cataract surgery. AL refers to the straight-line distance from the corneal apex to the fovea, reflecting the anatomical size of the anteroposterior diameter of the eyeball. The findings obtained from this investigation indicate that patients with longer AL had a significantly higher risk of developing PVD after surgery, suggesting that these patients may face a higher risk of complications during surgery. In clinical practice, an increased axial length (AL) is frequently observed in conjunction with high myopia, a condition that has been well-established as a major contributing factor for the development of PVD (21). For patients with high myopia, excessive AL may lead to choroidal thinning and reduced blood perfusion of the choriocapillaris (22), thereby causing a chronic low perfusion state at the retinal-choroidal interface, which may affect the structural stability between the vitreous and the retina, thereby increasing the risk of PVD.

Currently, phacoemulsification is the main surgical method for treating cataracts. Its principle is to decompose the cloudy lens nucleus into tiny particles through high-frequency ultrasonic vibrations, and then remove them through a suction system (23). However, the high-frequency mechanical vibrations, localized heat generation, and turbulence induced by irrigation-aspiration during this process can significantly disrupt the vitreous gel structure. Specifically, these physical effects undermine vitreous stability through three primary mechanisms: Firstly, acoustic microfluidics and cavitation effects generate transient shear forces at the vitreous base, causing disruption of the collagen-hyaluronic acid network; Secondly, localized temperature increases of 2–4°C accelerate hyaluronic acid depolymerization, thereby promoting vitreous liquefaction; Thirdly, prolonged irrigation operations induce pulsatile expansion of the vitreous cavity volume, exerting traction on the posterior cortical-retinal boundary membrane and diminishing its adhesive strength.

The findings of this study indicate that both cumulative ultrasound energy and surgical duration constitute independent risk factors for PVD. This observation aligns closely with the aforementioned pathophysiological mechanisms. Prolonged cumulative ultrasound exposure implies the ocular globe is subjected to heightened mechanical stress and thermal load, potentially inflicting impact-induced damage upon the vitreous base and posterior structures. This disruption of the vitreous gel state thereby elevates the risk of PVD occurrence. Prolonged surgical duration not only extends intraocular perfusion time but may also persistently disrupt the vitreous support system through frequent instrument entry into the anterior chamber and repeated traction on structures such as the suspensory ligament and ciliary body. This enhances mechanical shear forces at the interface between the posterior vitreous boundary membrane and the retinal inner limiting membrane, thereby further accelerating PVD formation. In view of this, to enhance both the safety and efficacy of the surgical procedure, it is essential to refine operative methods, reduce the duration of ultrasound energy application, and limit the total energy delivered, thereby minimizing disruptive biophysical effects on the vitreous cavity. Concurrently, improving the operator’s operational proficiency, optimizing the surgical process and reducing unnecessary tissue interference are of great significance for shortening the operation time, reducing the risk of intraocular trauma and postoperative complications.

Subsequently, a predictive model for postoperative PVD in cataract patients was established using five identified key variables (age, AL, preoperative vitreous liquefaction degree, CUE time, and surgical time), followed by performance analysis. High AUC values (0.963 and 0.955) were observed in both the modeling and validation groups, indicating the model effectively distinguished patients with PVD from those without. To prevent overfitting, the ratio of variables to events in the modeling group (5 variables, 80 PVD events) and the validation group (5 variables, 84 PVD events) conformed to the 1:10 rule. Concurrently, internal validation was conducted using the Bootstrap method (repeated 1,000 times), yielding post-validation C-index values of 0.963 and 0.955, respectively. This indicates robust model stability with no evidence of significant overfitting. This also indicates that the model exhibits strong predictive performance not only in the original development cohort but also maintains robustness and consistency when applied to external datasets, highlighting its reliability and generalizability across different populations. DCA demonstrated that the model can provide significant net clinical benefit when the risk threshold exceeds 0.02. This suggests that the prediction model can provide relatively accurate judgments for patient risk assessment, helping physicians achieve precise individualized treatment decisions in surgical decision-making.

This study’s nomogram model for predicting postoperative PVD in cataract surgery demonstrates significant innovation in indicator integration, tool format, and validation logic, echoing and differentiating itself from the recent development trends of ophthalmic AI prediction models. In terms of indicator dimensions, previous studies have largely been limited to single-type variables. For example, Kim et al., in constructing a predictive model for the progression of myopic normal-tension glaucoma, primarily relied on conventional clinical indicators such as intraocular pressure and axial length (24); while Koornwinder et al. used multimodal AI to fuse electronic health records and retinal nerve fiber layer scan data, they remained confined to the traditional framework of combining basic physiological parameters with imaging features (25). In contrast, this study systematically integrates preoperative physiological conditions (such as age, axial length, and preoperative vitreous liquefaction) with intraoperative operational factors (such as ultrasound energy accumulation time and total surgical duration), covering the complete mechanistic chain of PVD occurrence from pathological basis to surgical induction. This compensates for the shortcomings of existing vitreous complication prediction models that neglect the dynamic effects of surgery, and is more consistent with real clinical scenarios. Regarding model form and validation strategies, Kim and Koornwinder et al. have relied on complex AI algorithms, which, while improving accuracy, have also brought problems such as weak interpretability and high barriers to application at the grassroots level. This study, on the other hand, constructs a visual nomogram based on multivariate logistic regression. Physicians only need to sum the scores of each indicator to quickly assess an individual’s PVD risk, significantly improving clinical applicability while retaining the advantages of multidimensional data integration.

Although the present study offers certain empirical support, several constraints must be acknowledged and targeted in subsequent investigations. Firstly, the research was conducted at a single institution and involved a moderately sized cohort, which may restrict the generalizability of the results across broader or more diverse patient populations. To strengthen the robustness and external validity of the proposed model, future studies should prioritize multicenter collaboration, incorporate larger sample sizes, and adopt prospective study designs. Such efforts would help confirm the model’s consistency and performance across varied clinical environments. Secondly, while the current analysis accounted for a range of variables potentially influencing the onset of postoperative PVD, additional confounding factors may not have been fully captured. These include genetic predispositions, habitual lifestyle behaviors, and prolonged use of specific medications, all of which could play a role in the development of surgical complications. Hence, upcoming research should aim to broaden the scope of collected data by integrating a more comprehensive set of covariates, enabling a more systematic assessment of both biological and behavioral risk indicators. This approach would support the refinement of the current predictive framework, ultimately improving its precision and utility in clinical practice.

In summary, the individualized nomogram prediction tool developed in this study provides a valuable resource for guiding the prevention and clinical management of postoperative PVD in cataract surgery patients. This tool not only facilitates preoperative risk assessment but also offers precise decision support for postoperative management. By accurately evaluating preoperative risk indicators, it promotes the effective integration of these factors into clinical decision-making, enabling healthcare providers to design tailored intervention strategies and bespoke follow-up plans. For patients predicted as high-risk by the model, it is recommended that B-scan or OCT examinations be conducted within 1 week post-operatively to detect early signs of PVD. Additionally, strenuous exercise, bending over, and lifting heavy objects should be avoided within the first month post-surgery to reduce the risk of retinal tears or detachment. Concurrently, this model serves as an efficient preoperative communication tool, enabling patients to intuitively comprehend the potential risks of postoperative floaters or retinal complications. This enhances doctor-patient communication efficiency and patient treatment adherence. This personalized approach holds promise for improving patient treatment outcomes, optimizing clinical prognosis, and reducing the incidence of postoperative complications.

## Data Availability

The original contributions presented in the study are included in the article/supplementary material, further inquiries can be directed to the corresponding author.
